# Supplementary figures and images for: Aripiprazole reduces liver cell division
Source: PLoS One. 2020 Oct 26;15(10):e0240754. doi: 10.1371/journal.pone.0240754 (PMC7588089; doi:10.1371/journal.pone.0240754)

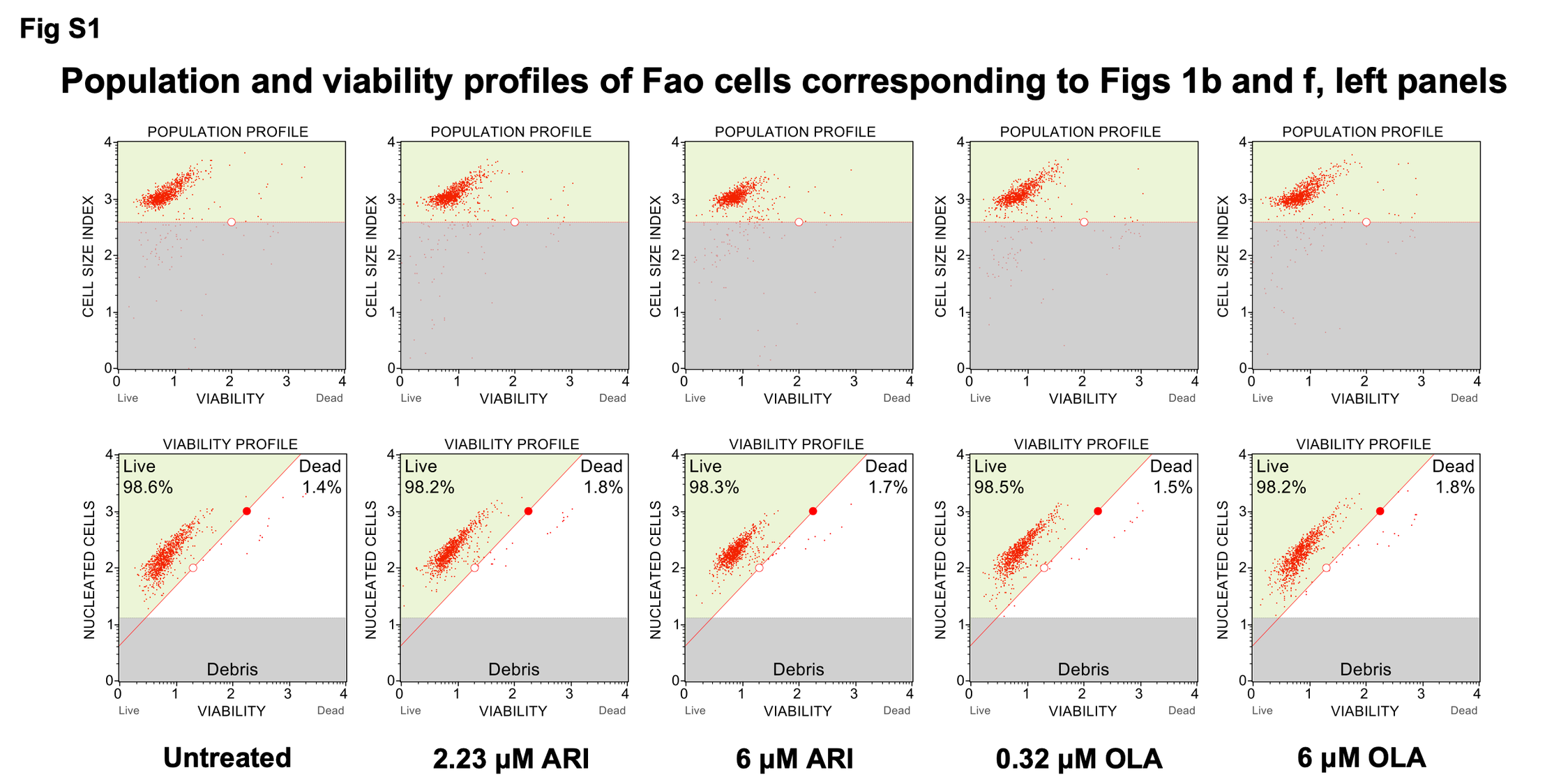

Supplement: S1 Fig — Population and viability profiles of Fao cells corresponding to Fig 1B and 1F, left panels. (TIF) [file pone.0240754.s001.tif]

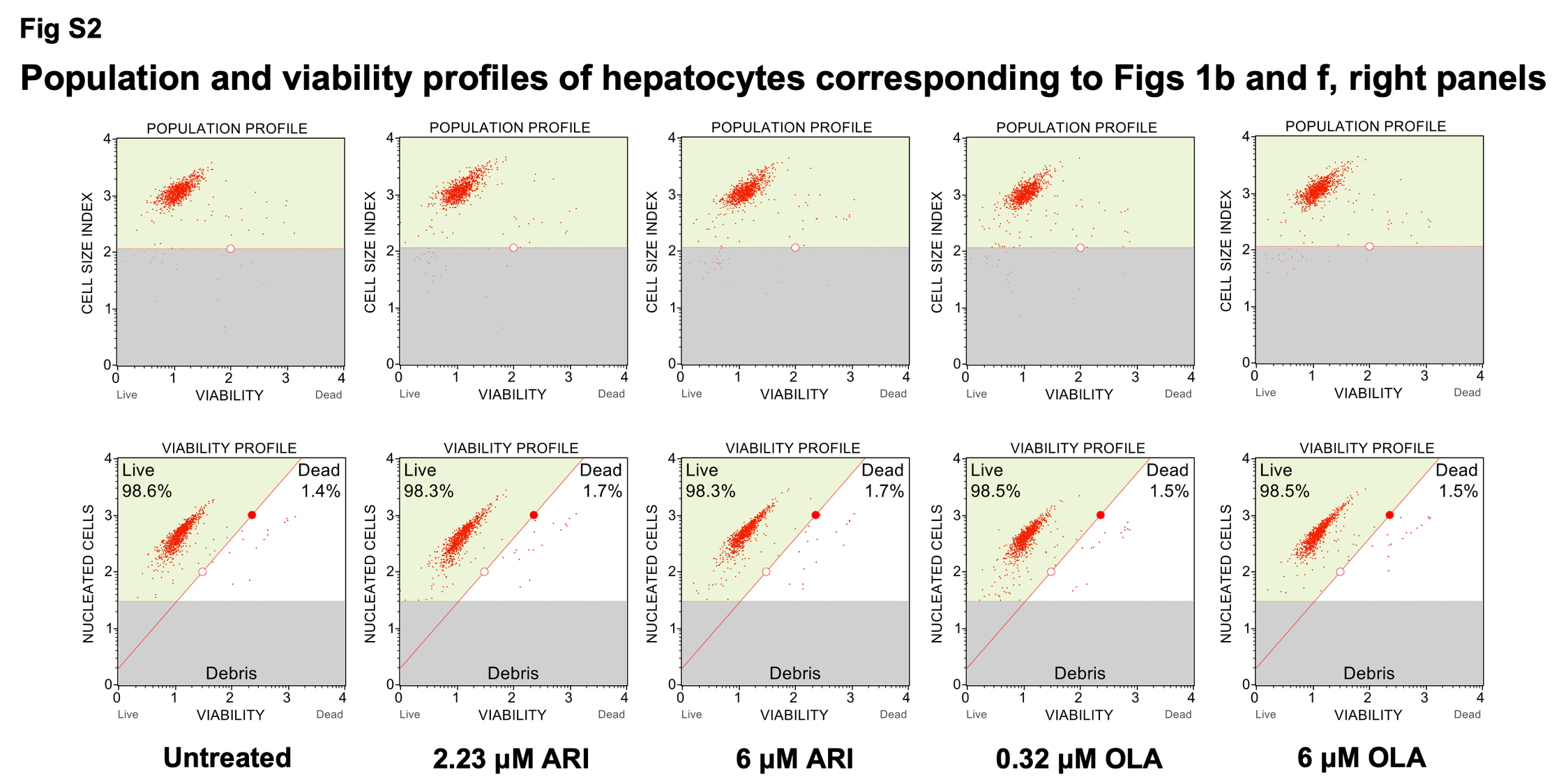

Supplement: S2 Fig — Population and viability profiles of hepatocytes corresponding to Fig 1B and 1F, right panels. (TIF) [file pone.0240754.s002.tif]

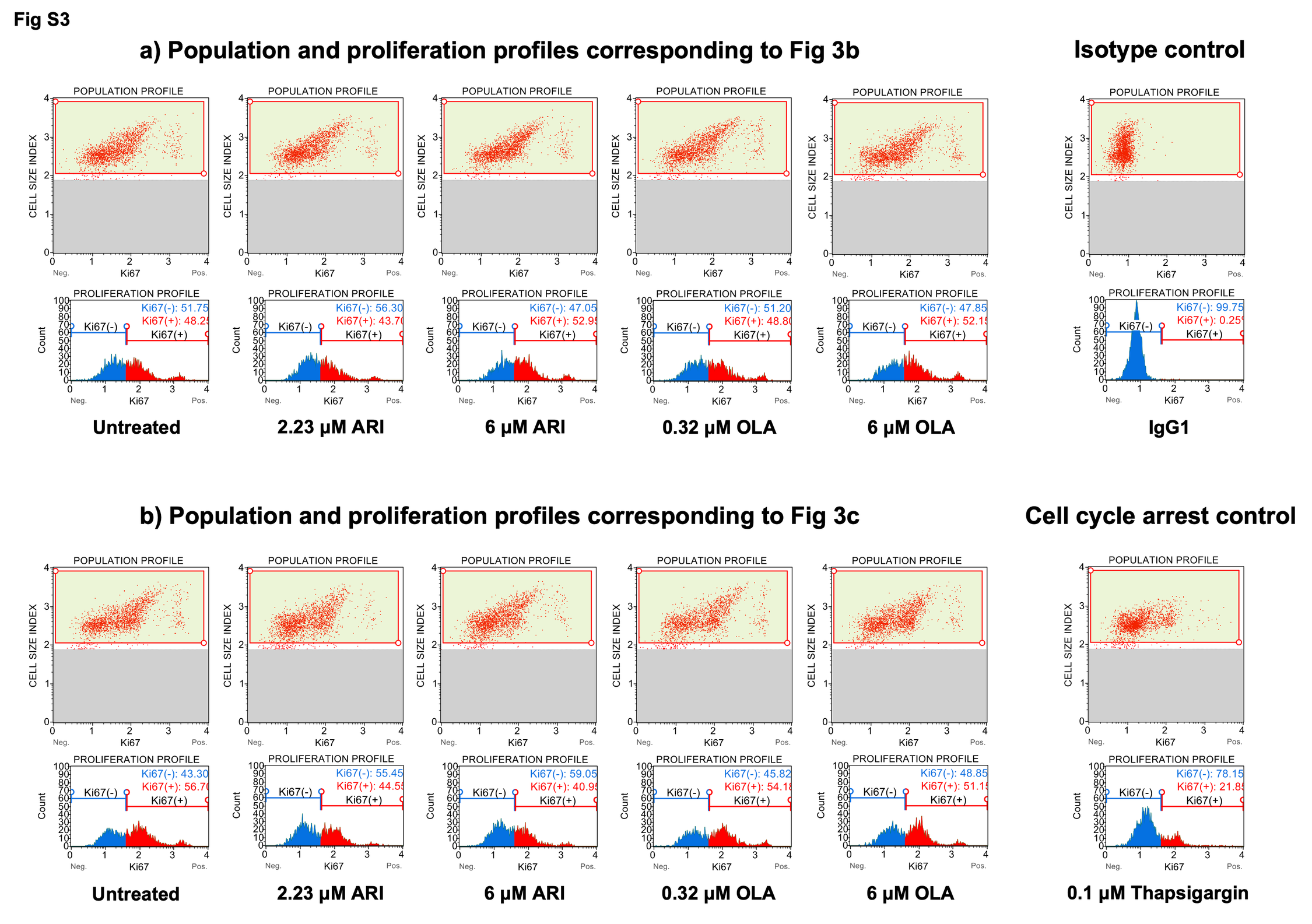

Supplement: S3 Fig — a) Population and proliferation profiles corresponding to Fig 3B. b) Population and proliferation profiles corresponding to Fig 3C. (TIF) [file pone.0240754.s003.tif]

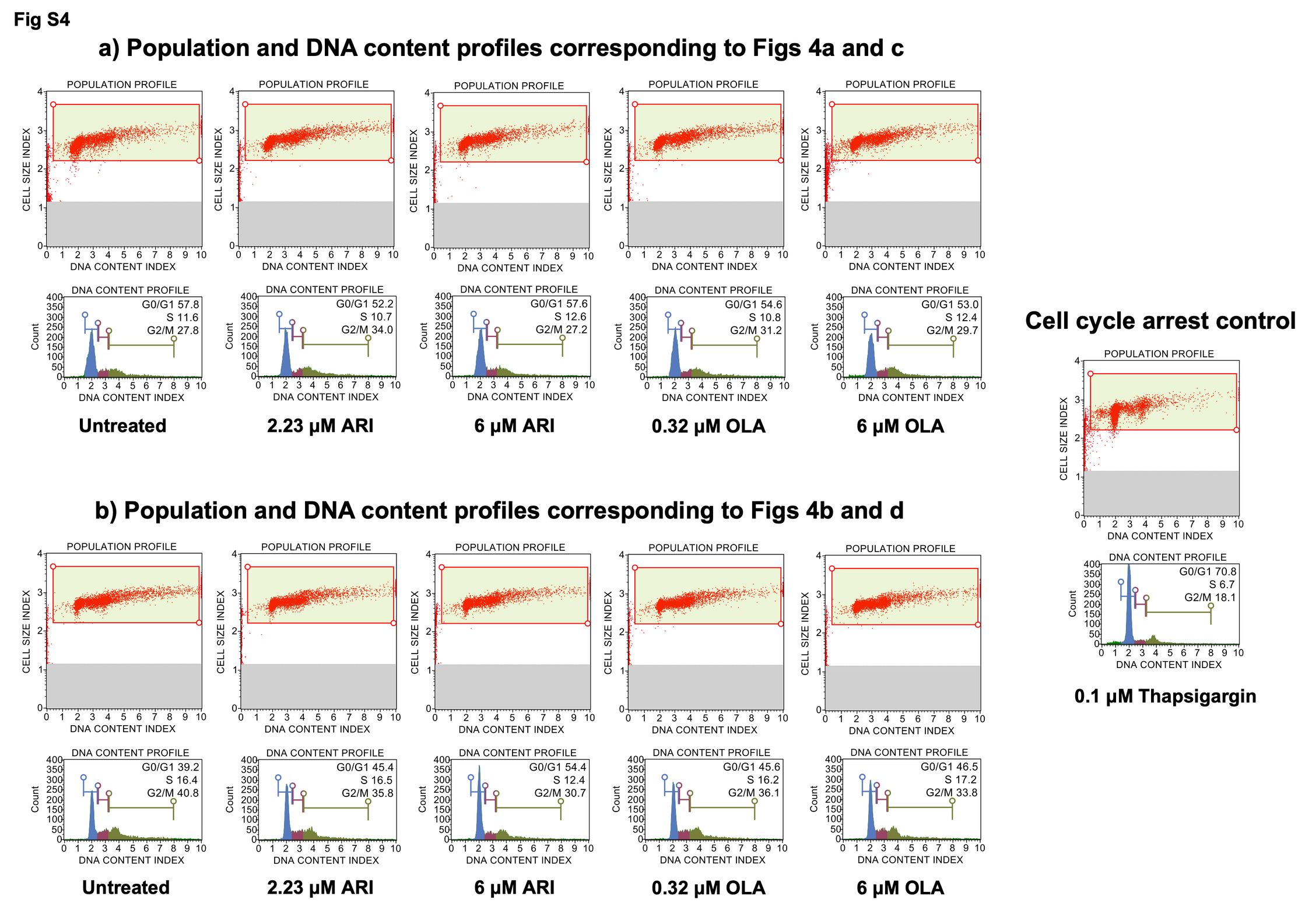

Supplement: S4 Fig — a) Population and DNA content profiles corresponding to Fig 4A and 4C. b) Population and DNA content profiles corresponding to Fig 4B and 4D. (TIF) [file pone.0240754.s004.tif]

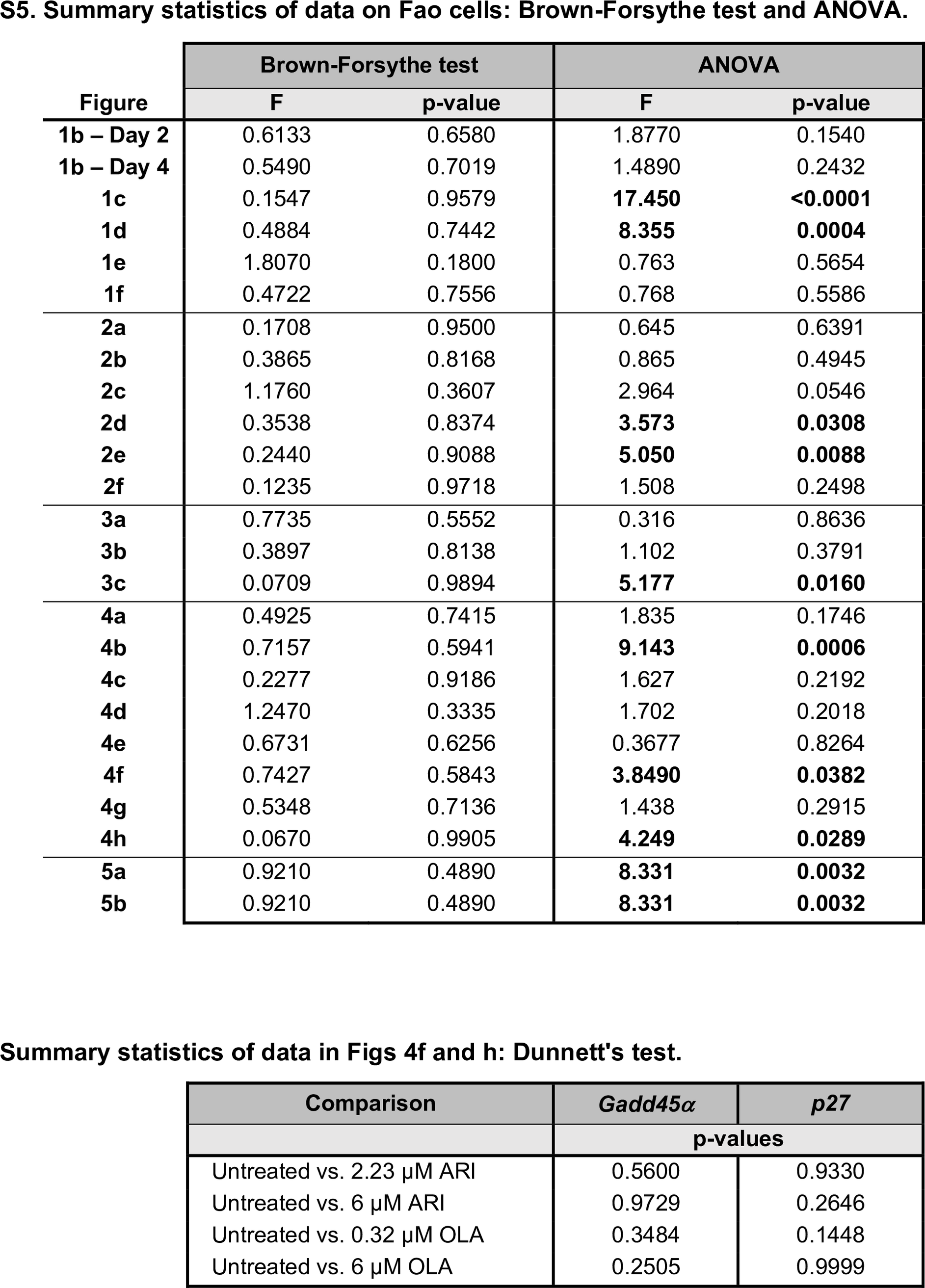

Supplement: S5 Fig — (TIF) [file pone.0240754.s005.tif]
